# Over-the-Counter Ibuprofen-Induced Pre-Pyloric Gastric Perforation in a 28-Month-Old Child: A Rare Pediatric Case

**DOI:** 10.7759/cureus.82821

**Published:** 2025-04-23

**Authors:** Wajeeh Uddin, Mariam Aylan Alshamsi, Vipul Gupta, Mohammed Alblooshi, Masih Abdul Kader

**Affiliations:** 1 Pediatric Surgery and Urology, Al Jalila Children's Specialty Hospital, Dubai, ARE; 2 Medicine, Dubai Health, Dubai, ARE; 3 General Surgery, College of Medicine, University of Sharjah, Sharjah, ARE; 4 Surgery, Tawam Hospital, Al Ain, ARE

**Keywords:** gastric perforation, ibuprofen, over-the-counter medication, pediatric acute abdomen, steroid therapy

## Abstract

Gastric perforation is a rare but potentially life-threatening cause of acute abdomen in pediatric patients beyond the neonatal period. Over-the-counter ibuprofen, often administered without physician oversight, can exacerbate gastric mucosal injury, particularly when used concurrently with corticosteroids. We describe the case of a 28-month-old male with bronchial asthma who developed acute epigastric pain, vomiting, and low-grade fever after receiving four doses of over-the-counter ibuprofen alongside prescribed oral steroids. Abdominal examination and investigations revealed generalized tenderness, elevated inflammatory markers, and pneumoperitoneum on radiographs. Diagnostic laparoscopy identified a pre-pyloric gastric perforation; however, tissue friability necessitated laparotomy and Graham patch repair. Postoperative recovery was uneventful, and further evaluation excluded hypergastrinemia and *Helicobacter pylori* infection. This case underscores the importance of thorough medication history-taking and highlights the need for heightened clinical suspicion of pediatric gastric perforation when multiple risk factors, such as the use of non-steroidal anti-inflammatory drugs and corticosteroids, are present. Prompt diagnosis, timely surgical intervention, and increased public health awareness of the risks associated with over-the-counter non-steroidal anti-inflammatory drugs in children are critical to reducing morbidity associated with this rare complication.

## Introduction

Acute gastric perforation is an exceptionally rare cause of acute abdomen in children beyond the neonatal period, with its precise incidence remaining incompletely delineated [1,2]. Although congenital gastric wall defects are commonly implicated in neonatal gastric perforations, the etiology in older pediatric populations is often multifactorial, encompassing infections (e.g., *Helicobacter pylori*), iatrogenic injury, trauma, or pharmacologic causes [3]. Among these, non-steroidal anti-inflammatory drugs (NSAIDs) have long been recognized as precipitators of peptic ulcer disease in adults; however, their role in pediatric gastric perforation is infrequently documented, underscoring the need for heightened clinical vigilance [4,5].

Over-the-counter ibuprofen, in particular, is readily available worldwide and is commonly administered to children without medical supervision [6]. When combined with other risk factors, such as concurrent corticosteroid therapy, this practice may increase the likelihood of gastrointestinal complications, including peptic ulcer disease and subsequent perforation [5]. Because the presenting symptoms of acute abdominal pain, vomiting, and hematemesis can mimic more prevalent conditions such as appendicitis, the diagnosis of NSAID-related gastric perforation in pediatric patients is frequently delayed, thereby contributing to significant morbidity and potential mortality [2,7].

In this report, we describe the case of a 28-month-old child who developed a pre-pyloric gastric perforation associated with the concomitant use of oral steroids and over-the-counter ibuprofen. The case highlights the importance of obtaining a thorough medication history in pediatric patients presenting with acute abdominal symptoms and underscores the need for increased public health education to mitigate the risks of unsupervised NSAID use in young children.

## Case presentation

A 28‑month‑old male child with bronchial asthma presented to the emergency department with acute epigastric pain and recurrent blood‑streaked vomiting that began on the morning of admission. The child had been on oral steroids for the preceding week because of an asthma exacerbation, in addition to inhaled steroids, bronchodilators, and a leukotriene receptor antagonist. Notably, his parents reported administering four doses of over‑the‑counter ibuprofen syrup over the past four days for generalized body aches. Additional symptoms included low‑grade fever and poor oral intake.

On examination, the child was febrile (38.3°C), tachycardic, and appeared dehydrated, prompting immediate intravenous fluid resuscitation. The abdomen was not overtly distended but displayed generalized tenderness and guarding. Laboratory investigations showed leukocytosis (WBC 24.6 × 10^9^/L), elevated C-reactive protein (68 mg/L), a procalcitonin level of 2.2 ng/mL, and electrolyte imbalances in the form of hyponatremia and hypochloremia (Table 1).

**Table 1:** Pertinent laboratory findings

Parameter	Patient Value	Reference Range (Age 2–5)
WBC (×10^9^/L)	24.6	6.0–15.0
CRP (mg/L)	68	<5
Procalcitonin (ng/mL)	2.2	<0.5

**Figure 1:**
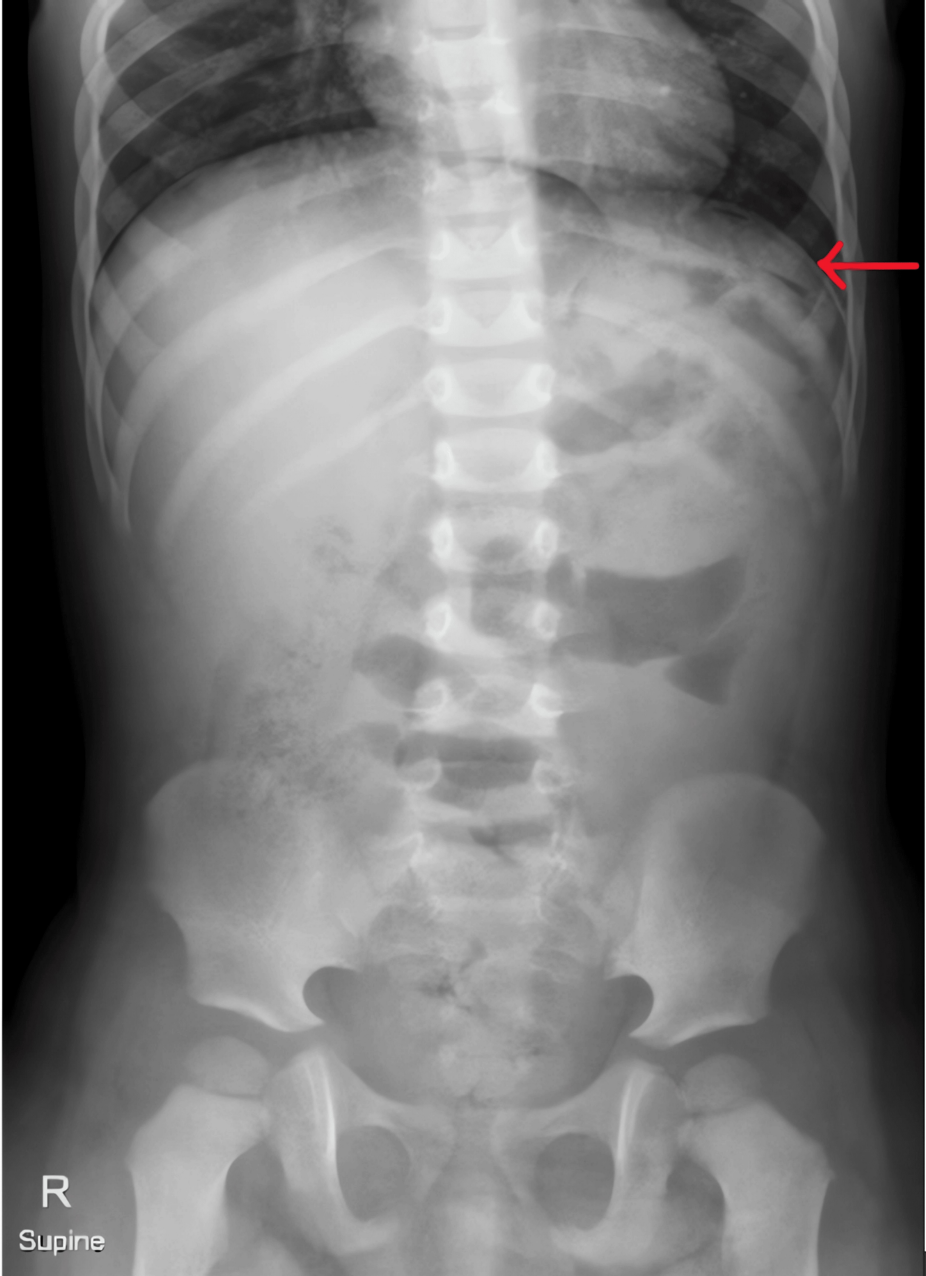
Erect abdominal radiograph showing pneumoperitoneum Subdiaphragmatic free air is evident, suggesting a perforation of a hollow viscus.

**Figure 2:**
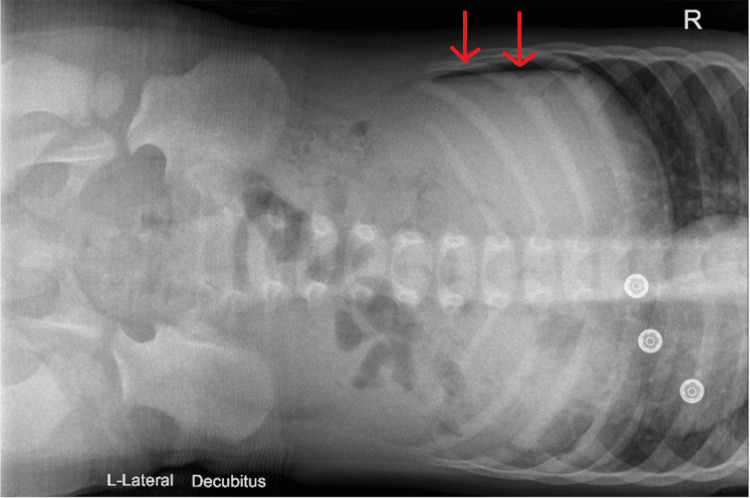
Cross-table lateral abdominal radiograph confirming free intraperitoneal air A small rim of free air is visualized (arrow), consistent with pneumoperitoneum.

Plain radiographs of the abdomen (Figures 1, 2) showed pneumoperitoneum, and an ultrasound scan revealed free fluid in the peritoneal cavity, with the appendix measuring 5 mm in diameter (within normal limits), leaving the exact source of perforation unclear. With a provisional diagnosis of gastrointestinal perforation, the child was taken for diagnostic laparoscopy following stabilization.

**Figure 3:**
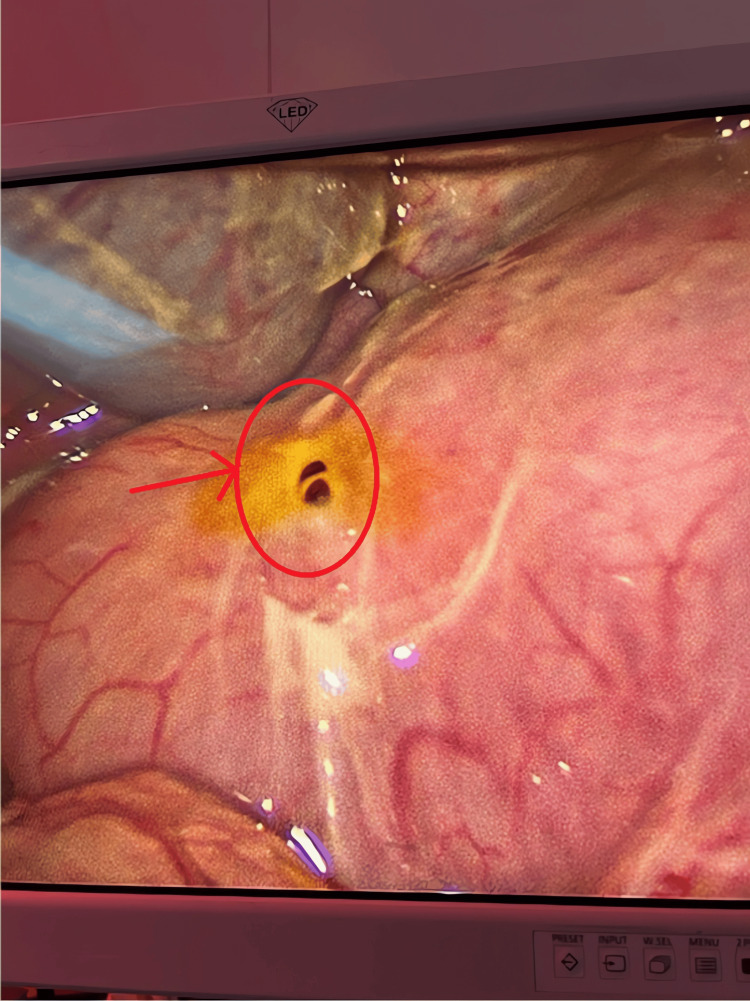
Intraoperative laparoscopic view of the pre-pyloric gastric perforation The perforation (arrow) is noted on the anterior wall of the stomach, with surrounding inflamed serosa.

Intraoperatively, there was bilious free fluid within the peritoneal cavity. The appendix appeared grossly normal, but a pre-pyloric perforation was identified on the anterior wall of the stomach (Figure 3). Because the perforation margins were friable, a laparotomy was performed, and the defect was repaired using a Graham patch. The postoperative course was uneventful, and the child was discharged in a satisfactory condition. Further evaluation, including serum gastrin levels and upper endoscopy with biopsy at eight weeks postoperatively, ruled out hypergastrinemia, *Helicobacter pylori* infection, or any other predisposing pathology.

## Discussion

Gastric perforation in the pediatric population remains a rare but clinically significant cause of acute abdomen, particularly beyond the neonatal period. While neonatal gastric perforations are frequently linked to congenital weaknesses or anomalies of the gastric wall, pediatric cases often reflect an interplay of multiple risk factors. In the present case, concurrent exposure to corticosteroids and over-the-counter ibuprofen appears to have culminated in a pre-pyloric perforation, underscoring the heightened risk of gastrointestinal complications associated with combined pharmacologic insults to the gastric mucosa.

NSAIDs are well-documented precipitators of gastroduodenal ulceration through cyclooxygenase inhibition, diminishing mucosal prostaglandin synthesis and weakening mucosal defense mechanisms. This effect is further potentiated by concomitant steroid therapy, which can impair mucosal healing and augment acid secretion. Although *Helicobacter pylori* infection remains a principal cause of peptic ulcer disease in adults, pediatric peptic ulcerations and perforations more often demand a careful medication history to detect iatrogenic or self-prescribed (over-the-counter) agents contributing to the pathology. Additionally, recent pediatric data underscore an increasing trend in NSAID-related gastropathy, with some investigations reporting a higher risk of peptic ulceration and perforation in children who use over-the-counter analgesics without medical supervision [8]. Concomitant corticosteroid therapy further exacerbates mucosal damage, highlighting the synergistic potential of these medications to compromise gastric integrity [9]. Although *Helicobacter pylori* remains an important contributor to ulcer disease, emerging evidence also emphasizes the role of medication-induced mucosal damage, particularly in regions with varying *Helicobacter pylori *prevalence [10].

Furthermore, a recent systematic review examining duodenal perforations presenting as acute peritonitis noted that peptic ulcer disease remains the predominant etiology, underscoring the significant role of ulcerative pathology in perforative complications [11]. While duodenal and gastric perforations differ in anatomic site, both highlight the importance of early recognition, especially when risk factors such as NSAID use are present.

A high index of suspicion is essential in diagnosing gastric perforation in children who commonly present with nonspecific symptoms such as acute abdominal pain, vomiting, and fever. Furthermore, the differential diagnosis routinely includes appendicitis, highlighting the need for diligent imaging studies, including erect or cross-table lateral radiographs to detect subdiaphragmatic free air. Early diagnosis is paramount, given the potential for rapidly evolving peritonitis, sepsis, and multi-organ dysfunction if management is delayed.

Surgical intervention whether laparoscopic or via laparotomy, remains the cornerstone of definitive management. Laparoscopy provides diagnostic clarity, enables limited peritoneal contamination through controlled lavage, and can allow primary repair or patch closure of perforations in select cases. However, friable tissue edges, as encountered in this patient, may necessitate conversion to open repair to achieve secure closure. The use of a Graham patch is a well-established technique for sealing perforations when edges are not amenable to primary approximation.

Finally, postoperative evaluation to exclude hypergastrinemia, *Helicobacter pylori* infection, or other rare causes of gastrointestinal perforation is advisable to guide longer-term management. While some rare etiologies, including dermatomyositis [12], trichobezoars [13], and gastric volvulus [14], have been described, the rising prevalence of over-the-counter NSAID use in the pediatric population warrants greater clinical vigilance. Early recognition, prompt surgical intervention, and comprehensive postoperative workup remain central to optimizing outcomes in these rare but serious presentations.

## Conclusions

This case underscores the importance of considering acute gastric perforation in children presenting with sudden onset of abdominal pain and features of peritonitis, particularly when there is a history of NSAID and steroid use. Thorough assessment of over-the-counter medication intake - often overlooked during routine history-taking - can be pivotal in early recognition and timely management. Given the potential for rapid clinical deterioration, a high index of suspicion, combined with prompt surgical intervention and diligent postoperative evaluation, is crucial to mitigating morbidity and mortality in pediatric gastric perforations.
